# Modified social ecological model: a tool to guide the assessment of the risks and risk contexts of HIV epidemics

**DOI:** 10.1186/1471-2458-13-482

**Published:** 2013-05-17

**Authors:** Stefan Baral, Carmen H Logie, Ashley Grosso, Andrea L Wirtz, Chris Beyrer

**Affiliations:** 1 Center for Public Health and Human Rights, Johns Hopkins Bloomberg School of Public Health, Johns Hopkins University, 615 N. Wolfe Street, Baltimore, MD 21205, USA; 2 Faculty of Social Work, University of Calgary, 2500 University Drive NW, Calgary, AB, T2N 1N4, Canada

## Abstract

**Background:**

Social and structural factors are now well accepted as determinants of HIV vulnerabilities. These factors are representative of social, economic, organizational and political inequities. Associated with an improved understanding of multiple levels of HIV risk has been the recognition of the need to implement multi-level HIV prevention strategies. Prevention sciences research and programming aiming to decrease HIV incidence requires epidemiologic studies to collect data on multiple levels of risk to inform combination HIV prevention packages.

**Discussion:**

Proximal individual-level risks, such as sharing injection devices and unprotected penile-vaginal or penile-anal sex, are necessary in mediating HIV acquisition and transmission. However, higher order social and structural-level risks can facilitate or reduce HIV transmission on population levels. Data characterizing these risks is often far more actionable than characterizing individual-level risks. We propose a modified social ecological model (MSEM) to help visualize multi-level domains of HIV infection risks and guide the development of epidemiologic HIV studies. Such a model may inform research in epidemiology and prevention sciences, particularly for key populations including men who have sex with men (MSM), people who inject drugs (PID), and sex workers. The MSEM builds on existing frameworks by examining multi-level risk contexts for HIV infection and situating individual HIV infection risks within wider network, community, and public policy contexts as well as epidemic stage. The utility of the MSEM is demonstrated with case studies of HIV risk among PID and MSM.

**Summary:**

The MSEM is a flexible model for guiding epidemiologic studies among key populations at risk for HIV in diverse sociocultural contexts. Successful HIV prevention strategies for key populations require effective integration of evidence-based biomedical, behavioral, and structural interventions. While the focus of epidemiologic studies has traditionally been on describing individual-level risk factors, the future necessitates comprehensive epidemiologic data characterizing multiple levels of HIV risk.

## Background

There is an increasing recognition of the importance of the social and structural drivers of acquisition and transmission of HIV [1,2]. While there is no singular definition, structural drivers can be conceptualized as those social, economic, organizational, and political power and domination factors which contribute to social inequities [2-4]. These structural drivers do not directly cause the acquisition or onward transmission of HIV; rather they mediate lower order risks such as those at the individual or network levels. Enhanced understanding of the various levels of HIV risk contributes to the recognition that HIV prevention measures must be delivered in the form of packages of services addressing multi-level HIV infection risks. As combination HIV prevention interventions focus on biomedical, behavioral and structural components, there is the need for a theoretical framework to guide the collection of data to characterize drivers of HIV risk at each of these levels [1,2].

Models may be used to visually represent theoretical explanations of biological, social and structural influences on disease processes, and can serve as useful guides for practice, research, intervention and policy development [4,5]. Some models have been used to articulate underlying individual motivations for behaviors, such as the health belief model, the theory of planned action, and the model of behavior change [6,7]. Other models, for instance the social ecological model, have functioned to situate health and health behaviors in the context of physical, social and policy environments [8,9].

Social ecological models are used to explain the complex associations between social (e.g. social networks) and structural (e.g. access to care) factors, individual practices, the physical environment and health [9]. The social ecological model contextualizes individuals’ behaviors using dimensions including intrapersonal (e.g. knowledge, attitudes, behavior), interpersonal/network (social networks, social support), community (e.g. relationships among organizations/ institutions), and public policy (e.g. local, state, national laws) to provide a framework for describing the interactions between these levels [10]. Ecosocial approaches employ biological and social analyses of population health to explore factors underpinning social inequalities and health disparities. Ecological models focusing on intrapersonal factors have been widely used in the design of effective interventions aimed at modifying individual behaviors [4]. However, few models have been developed to guide the measurement of individual level risks, both biologic and behavioral, as well as higher order levels of risk in the context of HIV infection.

The HIV epidemic can arguably be considered to be a group of interrelated epidemics, each with its own individual, social and structural risk factors. Sub-epidemics within populations have differing dynamics. No one model can describe all risk factors across these diverse domains. Conceptualizing epidemiological profiles from a social ecological theoretical stance therefore necessitates model specificity and contextual, multi-level analyses that incorporate social structure, social and community norms, and biological factors [5]. McLeroy [10] described: “one of the problems with many ecological models of social behavior is that they lack sufficient specificity to guide conceptualization of a specific problem or to identify appropriate interventions” (p. 355). To adequately describe and address the complexity of an epidemic such as HIV, unique and granular models can be developed for specific populations to measure relevant risks and risk contexts. After a comprehensive review of the literature, we found no model designed to date that encapsulates individual HIV transmission risks in the context of social and structural drivers of the epidemic. Auerbach et al. [1] developed a model to assess social and structural drivers of HIV to inform intervention development. Poundstone et al. [9] presented a heuristic framework of the social epidemiology of HIV that highlights the social and structural determinants of the epidemic. Other models have examined ecological-level risk factors for HIV such as structural violence [11,12] and social factors such as stigma and discrimination [4].

We build on past frameworks by a) examining multi-level risks and risk contexts for HIV infection and b) situating individual risks in the network, community, and public policy contexts as well as the epidemic stage. We developed the modified social ecological model (MSEM) to help visualize multi-level domains of HIV infection risks and guide the development of epidemiologic studies of HIV. We argue that data on risk factors and these multiple levels should be collected routinely as part of any epidemiologic study.

## Discussion

### The modified social ecological model (MSEM)

**Figure 1:**
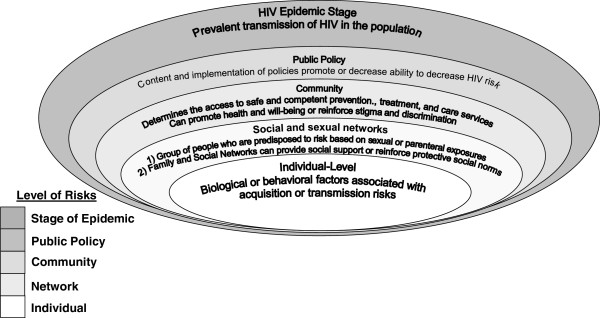
Modified social ecological model for HIV risk in vulnerable populations.

The MSEM is composed of five layers of risk for HIV infection: individual, network, community, policy, and stage of the HIV epidemic. The MSEM modifies the social ecological model by modifying the levels of risk as well as adding the stage or level of the HIV epidemic to the social ecological model, and is based on the premise that while individual level risks are necessary for the spread of disease, they are insufficient to explain population level epidemic dynamics. The higher order social and structural levels of risk (network, community, policy, stage of epidemic) represent risk factors outside of the control of any individual person [13]. And though policy makers tend to target interventions at individual level risks, they are only one component affecting the transmission of HIV among marginalized populations [2,4,9]. We present an MSEM figure that highlights these levels of risk (Figure 1) that can be adapted to contextualize HIV transmission risk among vulnerable populations. Factors can span levels and therefore the boundaries between levels may be understood as porous rather than distinct.

*Individual* factors are biologic or behavioral characteristics associated with vulnerability to acquire or transmit illness or infection [9,10]. These risks should be measured when there is biological or public health plausibility of being actual risk factors, ideally secondary to a rigorous systematic review with meta-analysis. While objective approaches to the synthesis of evidence for all levels of risk are preferred, in 2012, there is generally only sufficient levels of evidence for systematic reviews and meta-analysis of individual-level risk factors given the focus on this level of risk in the majority of epidemiological assessments of HIV.

*Social and sexual networks* are comprised of interpersonal relationships including family, friends, neighbors and others that directly influence health and health behaviors in multiple ways [10]. Networks, not bound by geography, socioeconomic status, or cultural, racial, or religious lines, include: “the webs of human relationships (including dyadic, familial, social, sexual, and drug-using) through which social (including sexual) exchange occurs and social norms are played out” [2, p. 2]. HIV risks—and other health outcomes—are associated with social influence, social engagement, disease prevalence, access to information, intimate contact and social networks [9]. In the MSEM, networks are operationalized as a group of people who have a higher probability of exposure to infectious disease from each other mediated through sexual exposure, shared use of injection, and/or non-injection drug paraphernalia, or increased physical contact. Sexual and social network levels of risk include biologic (e.g. HIV infection rates) and behavioral (e.g. sexual contact, shared use of injection drug paraphernalia) factors that potentiate HIV transmission among individual members of a network [14,15]. Alternatively family and social networks can provide social support and reinforce social norms and behavior that serve as protective factors and reduce HIV transmission risks [15]. The measurement of network-centric data in HIV epidemiological studies is crucial given how determinative network characteristics are in predisposing or protecting individuals within those networks to the acquisition and transmission of HIV.

*Community* environments can either promote health and well-being or be a source of stigma. The definition of who and/or what constitutes a ‘community’ is contested but generally includes: network ties; relationships between organizations and groups; and geographical/political regions [10]. Cultural, economic, religious, geographic lines, prison walls, or any combination of the above may bind communities. Urban, neighborhood, or community deprivation and disadvantage can increase vulnerability to HIV [16]. Socio-cultural norms and values, social cohesion and network structures are shaped by larger social-structural forces and influence interpersonal processes and individual behaviors [1,13]. Interpretation of community norms may increase or mitigate the risk level for HIV infection within the community. For example, interventions focused on establishing condom use norms have demonstrated efficacy in increasing condom use [13,17]. Stigma affecting populations at risk for the acquisition and transmission of HIV often manifest at the level of the community in limiting the provision and/or uptake of HIV prevention, treatment, and care services.

*Laws and policies* of any state provide the general framework for shaping the risk of marginalized populations as well as the general population [18]. These policies and their financing [19] and implementation either promote or decrease the community’s ability to provide preventive or harm reduction services (e.g. needle exchange; condom provision in prisons) to its constituents by passing laws making such actions legal or illegal or by providing or disrupting funding mechanisms supporting these programs [13,20,21]. Legal and policy environments play a critical role in hindering—or supporting—HIV prevention programs among sex workers [22]. There are numerous examples worldwide of laws-such as criminalization of homosexuality, sex work and substance use, or criminalization of prevention practices, such needle exchange or methadone assisted treatment (MAT)—founded in morals, cultural relativism, and political will rather than the results of public health science. In such contexts, although marginalized populations such as sex workers, people who inject drugs (PID) and men who have sex with men (MSM) have elevated HIV infection risks there are a lack of scientifically proven targeted prevention and harm reduction strategies [22]. Policies determine allocation of economic resources to education, health care, job training, financial assistance and HIV prevention services and therefore play a substantial role in shaping structural contexts of HIV risk [13]. Downstream, these laws and policies likely impact networks; for example, incarceration can both disrupt and create new networks. Similarly, policies can drive conflict and economic disruption affecting the provision and uptake of services, the makeup of social and sexual networks. Often the highest impact of such adverse effects are on populations already marginalized [23].

Ultimately it is the *stage of the epidemic* within the social and sexual network, community, and country that will determine the risk of disease acquisition for the individual [13,24]. No behavior, policy or law, community determinant, network attribute, or individual characteristic can create infectious disease; rather these can only create conditions which either increase or decrease the probability of acquisition or onward transmission of an already prevalent disease. The stage of the epidemic can be quantified in several ways including HIV incidence and HIV prevalence. In the context of populations with high prevalence of HIV, mean and total community viral load has been used as a marker of population-level transmission of HIV. Thus, the risk associated with any individual practice such as unprotected anal intercourse should be interpreted within the context of the stage of the epidemic as the risk of this practice should be considered as high-risk only in the context of a high burden of HIV infection and viral load.

We present two case studies to demonstrate the use of the MSEM in enhancing understanding regarding the multifactorial, multilevel infection risks of different HIV epidemics: 1) parenteral transmission of HIV among PID; and 2) sexual transmission of HIV among MSM.

### Case study 1: Parenteral transmission of HIV among people who inject drugs (PID)

**Figure 2:**
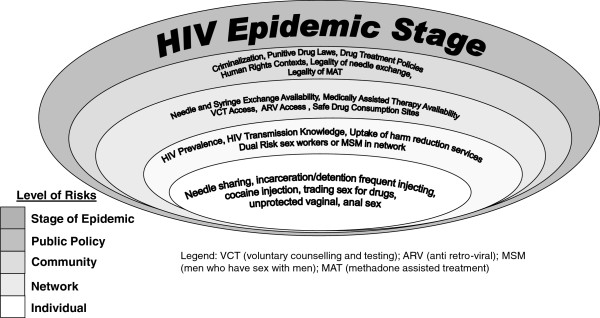
Modified ecological model for HIV risk in people who inject drugs.

Approximately 20.0%, or 3 million, of PID are living with HIV across the globe [25]. There is a wealth of literature devoted to the *individual*-level risk factors for HIV acquisition and transmission among PID (Figure 2). HIV infection has been associated with: the duration and frequency of drug use, injecting practices (e.g. ‘jerking’, ‘scaling’, backloading, etc.), drug injection location, co-infections (HCV, HBV, sexually transmitted infections [STI], genital ulcerative disease[GUD]), sexual risk factors (e.g. unprotected receptive anal intercourse, frequency and number of sexual partners), type of drug used (e.g. poppers, meth), marginalized groups (e.g. Black and minority ethnic populations [BME]), psychiatric comorbidities, sharing drug use paraphernalia as well as other non-drug use related risks including tattooing, blood transfusions and organ and tissue transplants [21,26-28] .

The *network* risk factors that drive the spread of HIV predominantly through moderation of these individual level risk factors are less appreciated. Networks of people who use drugs include social networks, injection networks of people with whom the person injects drugs, and sexual networks. Sexual/injection networks are more proximal in the exposure to HIV, though social networks may provide differential effects depending on the health literacy of members. Social and peer-group norms, population mobility, drug costs, exposure and access to drugs, sexual roles (e.g. receptive or insertive intercourse), availability of condoms in networks, and high HIV/STI prevalence in social/sexual networks can result in transmission of HIV and other co-infections such as HCV [27]. Higher risk injection practices among heroin injecting networks, such as sharing contaminated drug solutions and needles, are more common in unsafe social environments (e.g. visible areas with limited privacy and/or security) [29]. In addition, accurate knowledge of HIV characteristics and the prevalence of injection and unprotected sex within social and/or sexual networks of PID contribute to the rate of HIV spread [21,27].

Relevant features of HIV prevention for PID at the *community* level include access to evidence-based harm reduction strategies such as needle and syringe programs (NSP), methadone maintenance programs, community health centers, safe injection sites, HIV education and preventive services, voluntary counseling and testing (VCT), health literacy, and access to antiretroviral therapy (ARV) [21]. Community-based services and community advocacy, engagement, and mobilization, in conjunction with a strong civil society and peer initiatives can address and reduce HIV risk among PID; conversely stigma, discrimination and marginalization of drug users exacerbates HIV risk [21,28]. Inequitable social norms contribute to HIV risks among PID who are: women, younger, sexual minority, and/or Black and minority ethnic (BME) populations [21,27,30]. For example, unbalanced power relations with male partners limit women’s ability to negotiate both safer sex and refusal to share needles [27].

The legality of many of the aforementioned harm reduction strategies is determined at the level of *public policy*. Policies determine a range of risk exposures for PID, including coverage of NSP, substitution therapies, drug treatment, HIV testing and counseling, sexual health education, criminalization of PID, condom availability, ARV access, drug trafficking routes, inclusion in national HIV surveillance, and police surveillance—all salient factors in shaping HIV risk among PID [20,21,27]. Less than one-third of PID globally have access to HIV prevention services and approximately 5% have access to NSP [20,21]. Access by PID to these services is greatly diminished, if not non-existent, when the provision of these services is criminalized. Furthermore, highly punitive drug laws resulting in frequent incarceration of PID also plays a role in propagating spread of disease by both limiting the access to harm reduction strategies and by concentrating uniformly high risk individuals in the same network [27]. Shifting the focus to human rights contexts, advocacy and drug treatment from detention, as well as provision of NSP and treatment in prisons, is therefore key to HIV prevention [20,21,28]. Income generation and employment programs, social housing, and access to free harm reduction materials (e.g. condoms, syringes) are examples of programs that can reduce HIV risk among PID [21,27]. Again, each of these factors is contextualized by the stage of the HIV epidemic and HIV prevalence in any particular setting, underscoring the need for country and population specific approaches [20,21,28].

### Case study 2: Sexual transmission of HIV among MSM

**Figure 3:**
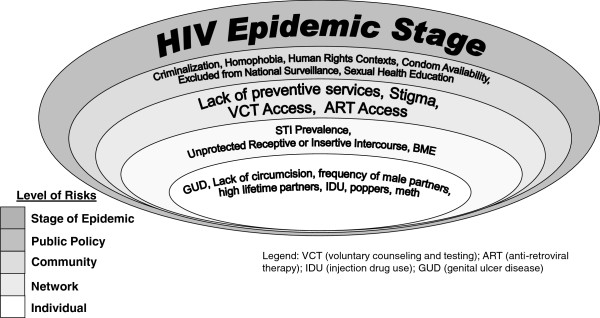
Modified ecological model for HIV risk in men who have sex with men.

In all settings where MSM have been studied, there is a disproportionate burden of HIV among MSM when compared to other men [31,32]. Sexual transmission risks among MSM are significantly shaped by inequitable social and structural contexts that influence individual’s sexual practices and access to HIV prevention [4,31] Figure 3 presents the levels of risk faced by MSM and some of the risk factors present at each level.

Among MSM, *individual* level acquisition risks have focused on the highest probability exposure: unprotected anal intercourse (UAI), and specifically on correlates of receptive anal intercourse [33]. Use of party drugs such as methamphetamines and alkyl nitrates (poppers) has been associated with heightened sexual exposure among MSM in several settings [33,34]. And as with men who only report sex with women, HIV transmission in MSM is associated with genitourinary disease, being uncircumcised, high frequency of male partners, and high lifetime number of male partners [15,35]. For those living with HIV, the biggest determinant of onward sexual transmission of HIV is the viral load of the insertive partner.

Social and sexual *network* level factors include the density and size of networks; these shape HIV risk for its members [36]. Larger networks provide increased opportunities for exposure to varied sexual practices and HIV positive potential partners [36]. Larger sexual networks have also been associated with increased reporting of unprotected anal intercourse among MSM in several settings [37,38]. Using phylogenetic methods, studies have consistently shown that HIV is transmitted in episodic bursts of transmissions among MSM. Moreover, sexual networks determine risk, particularly among sexual networks configured of MSM with higher rates of sex work, drug use and accessing the internet for sexual partners [14,15,39]. Separately, higher risk networks including MSM with higher viral load related to undiagnosed HIV infection, acutely infected MSM, or those with late stage infection HIV infection. High prevalence of infections causing genital ulcerative disease will increase the probability of HIV transmission within networks. Interpersonal skills training with MSM regarding safer sex negotiation was associated with reduced UAI [40] as were interventions focused on promoting condom use within social networks [17].

*Community* norms and values that stigmatize same-sex practices and sexually diverse populations present significant barriers to accessing HIV prevention among MSM, as well as access to other health care services [2,4,31,41,42]. Stigma in communities limits coverage of services by limiting both the provision and uptake of HIV prevention, treatment, and care services. Provision is limited through limited funding for these services and limited legality and willingness of entities to provide services. Even when services are provided, coverage is limited by reduced health seeking practices and utilization of health and HIV services among MSM, due to fear of disclosure and discrimination, may reduce knowledge of UAI risks and access to prevention methods (e.g. condoms, lubricant). Sexually diverse populations face widespread social exclusion from families, friends, cultural, religious and health institutions which inhibit disclosure of sexual orientation and/or HIV-positive serostatus and play a key role in exacerbating HIV risk [31]. Other stigma and discrimination not related to sexual practices, may also elevate risk, for example, BME MSM in developed country settings have higher HIV infection risks in comparison to Caucasian/white MSM [43], highlighting the importance of understanding the role intersecting forms of social and structural discrimination (e.g. racism, homophobia) play in shaping health outcomes and risk [4].

Public *policies* such as the criminalization of homosexuality in more than 80 countries and exclusion from national surveillance programs are, to some extent, to blame for the dearth of targeted, accessible prevention strategies for MSM and thus continually increasing global incidence rates of HIV [31,41,44,45]. The vast majority of MSM globally do not have access to HIV prevention, treatment and care services [42,46]. Discrimination of sexual minorities by police and health care providers are global phenomena: anti-discrimination training and policies are therefore imperative to protect human rights and promote health [44,47-49]. Ultimately, the act of men having sex with men is not inherently dangerous; in fact, only in the context of an advanced stage of the epidemic among MSM and lack of preventive services (or awareness/uptake of services) are actually risk exposures for HIV infection [50]. The porous nature of these levels should also be considered; while receptive anal sex (individual risk) poses higher HIV infection rates this in fact occurs in a dyadic process (network) influenced by socio-cultural norms (community).

## Summary

This paper has proposed a model to guide epidemiologic studies of HIV in collecting the data needed to enhance characterization of multi-layered risk contexts. The modified social ecological model functions as a useful framework with which to characterize and visualize the various layers of risks for HIV. The model includes five levels of risk: individual, network, community, public policy, and stage of epidemic. Each level provides a context in which to understand subsequent levels and there is interaction between each level and factors within levels. Other than epidemic stage, each of the levels function as targets for prevention strategies. One of the unique challenges of conceptualizing a model for infectious diseases is the porous nature of these levels. The flexibility of the model was demonstrated by describing two contemporary HIV epidemics: transmission of HIV among PID among MSM, though the model could be adapted to understand risks faced by other populations.

Behavioral and biomedical interventions tend to focus on decreasing individual and network level risks of HIV. However, the effectiveness of these interventions as measured by reductions of HIV incidence will ultimately be limited by the community, public policy, and epidemic stages in which they are operationalized. To date, the majority of evaluations of biomedical and behavioral interventions have focused on efficacy rather than real-world effectiveness. Moving forward, there has been a renewed emphasis on implementation science to assess the effectiveness of interventions. There appears to be consensus that translating efficacious interventions to effective programs necessitates addressing higher order risk factors. However, to date there remains a limited evidence base in the peer-reviewed literature supporting structural interventions. Moreover, the interventions or programs attempting to change community dynamics such as stigma or public policy are more difficult to implement and evaluate than individual-level interventions amenable to rapid scale-up and blinded randomized trials [51]. Similarly, new approaches are needed for the evaluation of evidence supporting such interventions transcending randomized controlled trials [52].

Ultimately, defining and characterizing individual level risks of HIV transmission is imperative in better understanding the dynamics of an epidemic. However, it is the higher order social and structural level of risks that likely facilitate HIV transmission on a population level. Simply said, it no longer contributes to our understanding of HIV to characterize that higher numbers of sexual partners, lower levels of condom use, and the sharing of drug injecting devices are associated, causally or not, with HIV infection. Ensuring that every epidemiologic study for HIV also characterizes social and structural factors that underlie high risk practices will likely result in far more actionable data in furthering the HIV prevention sciences.

## Competing interests

The authors declare that they have no competing interests.

## Authors’ contributions

The idea of developing a social ecological framework to guide epidemiological studies was developed by SB. SB and CB conceptualized and led the writing of the paper. CL conducted a literature review, helped refine the model, and wrote significant portions of the manuscript. All authors contributed to the conceptual development of the model, supported writing sections of the manuscript, critical revision of the manuscript, and approved the final version.

## Pre-publication history

The pre-publication history for this paper can be accessed here:

http://www.biomedcentral.com/1471-2458/13/482/prepub
